# Supplementary material for: The CYP71A, NIT, AMI, and IAMH gene families are dispensable for indole-3-acetaldoxime-mediated auxin biosynthesis in Arabidopsis
Source: Plant Cell. 2025 Oct 15;37(11):koaf242. doi: 10.1093/plcell/koaf242 (PMC12586335; doi:10.1093/plcell/koaf242)
Supplement: koaf242_Supplementary_Data [file koaf242_supplementary_data.zip › Supplementary Figures.docx]

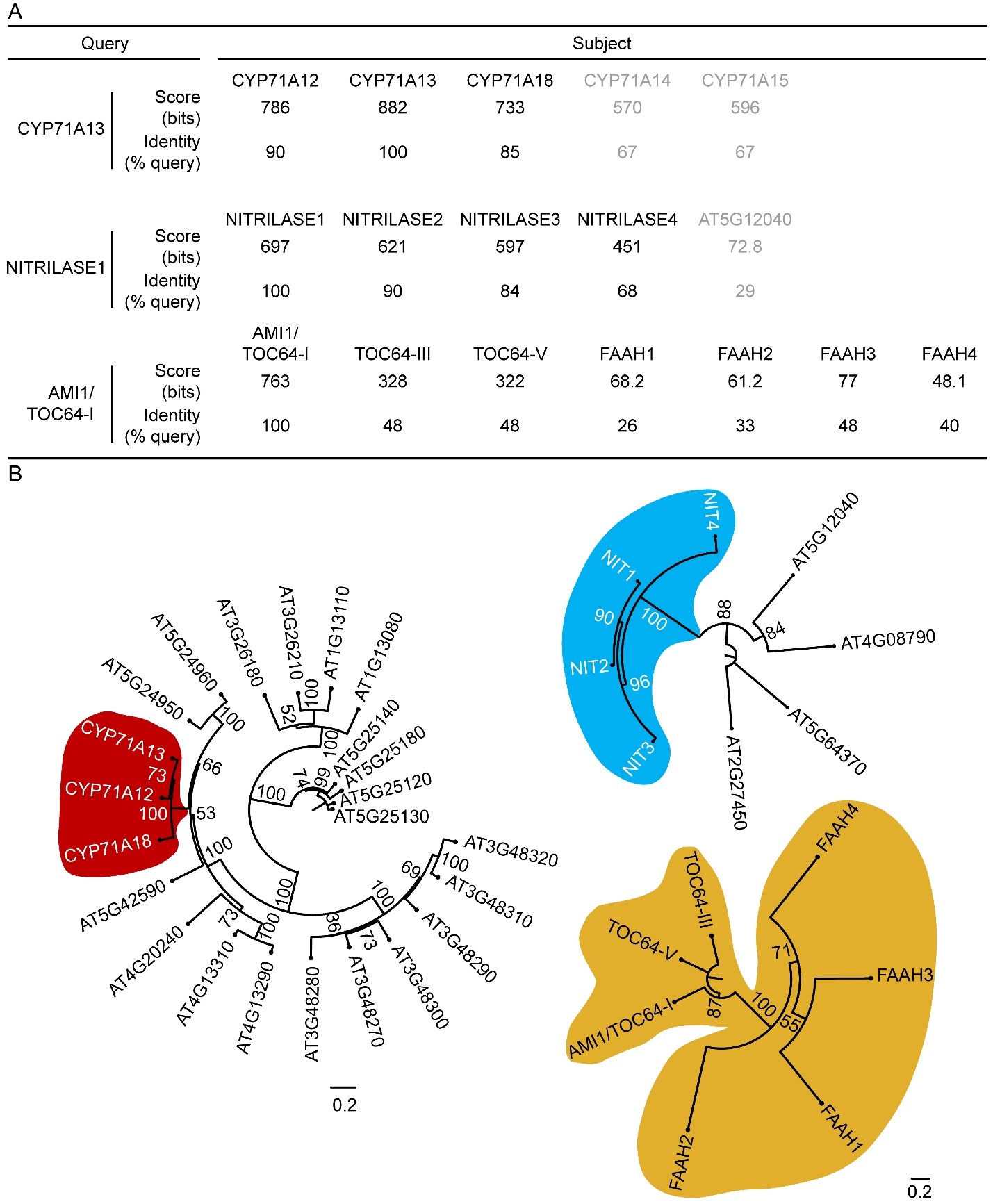


**Supplementary Figure S1**. **Phylogenetic comparison of amino acid sequences of Arabidopsis CYP71A, NIT, and AMI1/TOC64/FAAH family members**. (A) Protein identity based on amino acid sequence comparison using protein basic local alignment search tool (BLASTP; Camacho et al., 2009). Marked in gray are genes not considered for this study due to their lower identity score to the founding member of each family. (B) Maximum likelihood phylogenetic trees made using MUSCLE algorithm in PLAZA Dicots 5.0 (Van Bel et al., 2021). Node numbers represent bootstrap values.


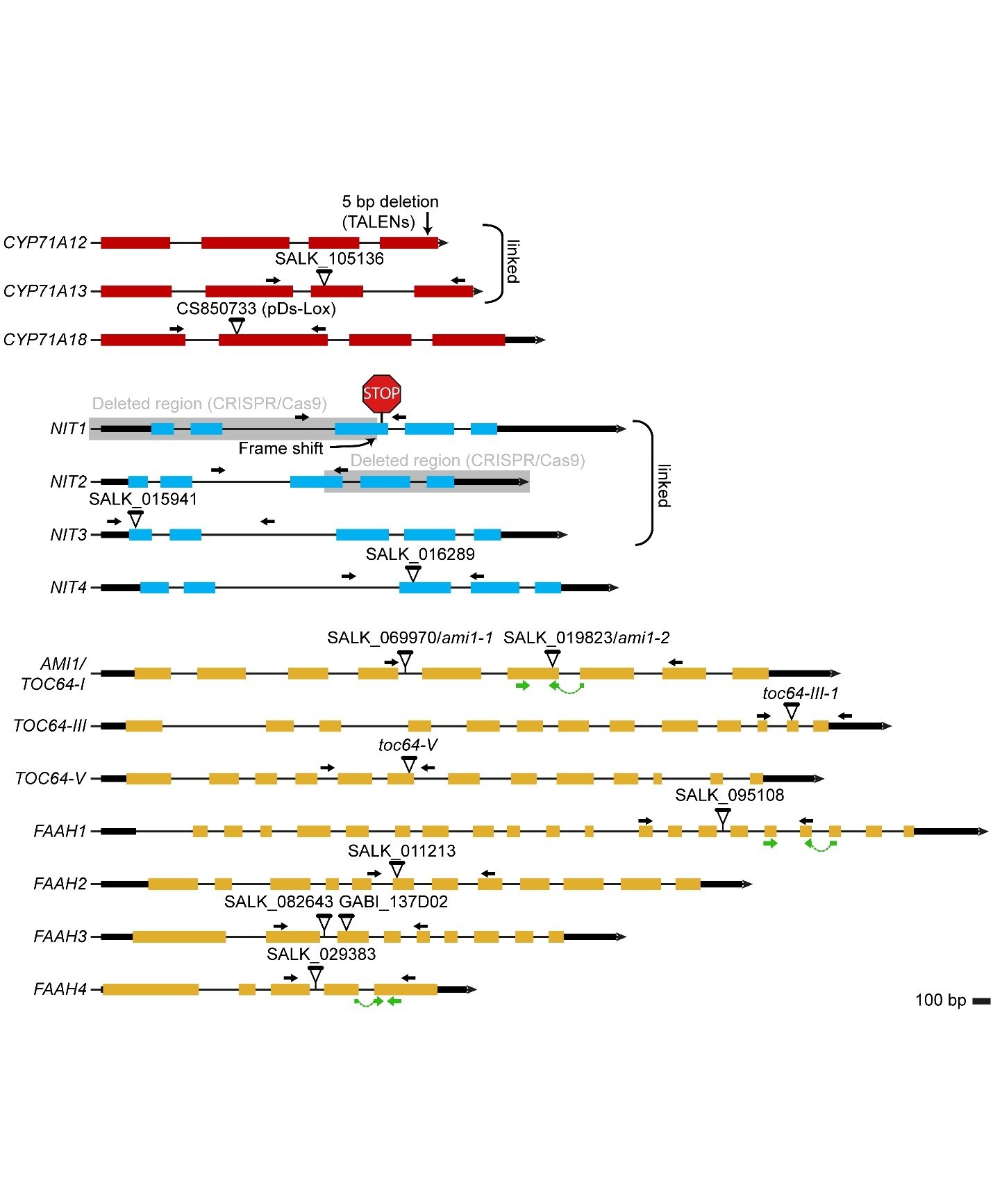


**Supplementary Figure S2**. **Schematic representation of gene structure (5’ to 3’) of *CYP71A*, *NIT*, and *AMI1/TOC64/FAAH* gene families examined in this study**. Colored rectangles mark exons. Black rectangles denote 5’ and 3’ untranslated regions (UTRs). Black lines represent introns and the arrowhead at the right end of each gene model indicates the 5’-3’ orientation. T-DNA insertions (triangles) and primer annealing sites for genotyping (black arrows) and qPCR (green) are shown. Grey background in *NIT1* and *NIT2* corresponds to deleted DNA sequences in the CRISPR line.


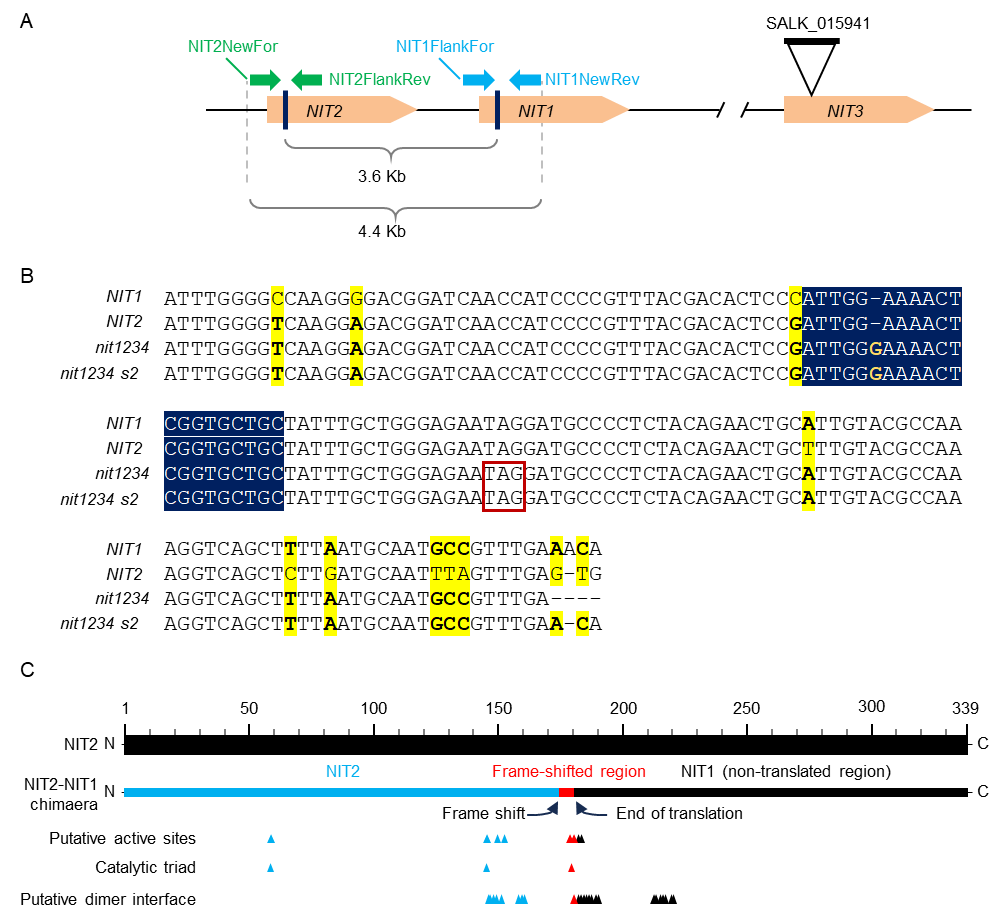


**Supplementary Figure S3**. **Genome editing of tandem *NIT2* and *NIT1* genes**. (A) Relative positions of two genetically linked *NIT* genes, *NIT2* and *NIT1.* Distances between the two gRNA target sites (3.6 Kb), locations of PCR genotyping primers (colored arrows), and the size of the PCR fragment in WT background (4.4 Kb) are indicated. (B) Chimeric frameshifted *NIT2-NIT1* gene resulting from CRISPR/Cas9 genome editing. The gRNA targeting site (navy blue background) is the merging point between the two genes and contains a single G insertion that leads to a premature STOP codon (red rectangle). Polymorphisms between *NIT1* and *NIT2* (yellow background) show that upstream of the merging point, the PCR-amplified chimeric fragment contains *NIT2*-like polymorphisms, whereas downstream of the merging point, polymorphisms are *NIT1*-like, which suggests a complete deletion of the 3.6 Kb fragment depicted in panel A. An addition of a single G at the junction site shifts the reading frame and makes the chimeric gene non-functional. *NIT1* and *NIT2* sequences were obtained from TAIR. Chimeric sequences from *nit1234* and *nit1234* *s2* were obtained by Sanger sequencing of the PCR fragment amplified using primers NIT2NewFor and NIT1FlankRev (primer sequences available in Supp. Table 3). *nit1234: nit1 nit2 nit3 nit4, s2: sur2.* (C) NIT2-NIT1 chimeric protein schematic showing the active sites predicted for NIT2 that are maintained (cyan) and lost (red and black) after the frame shift caused by the insertion of an extra G, as depicted in panel B. NIT2 amino acid residues that belong to putative active sites, catalytic triad, or putative dimer interface were obtained from the conserved domain database (Wang et al., 2023) accessed through BLASTP (Camacho et al., 2009) at the National Center for Biotechnology Information website (https://blast.ncbi.nlm.nih.gov/Blast.cgi).


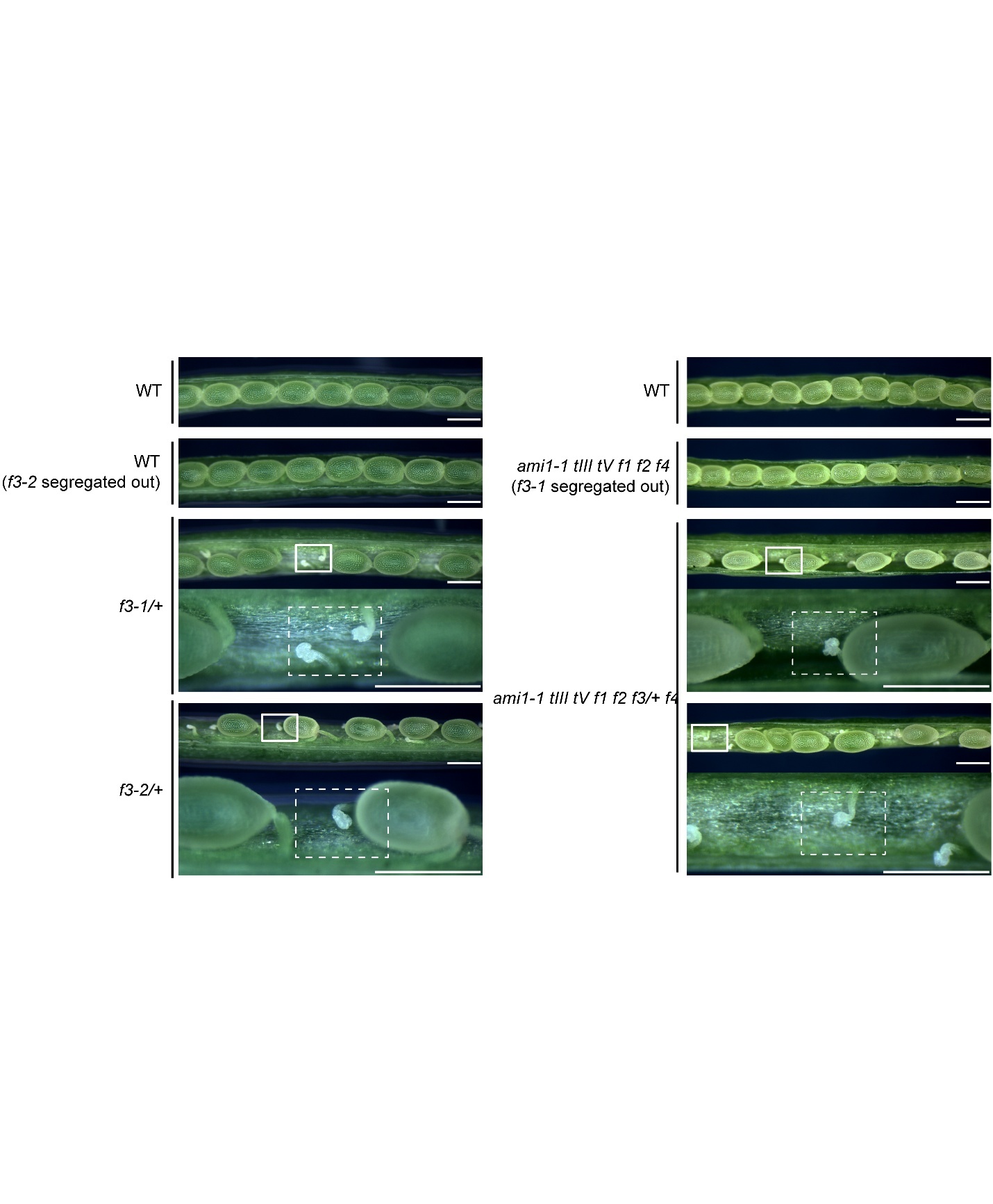


**Supplementary Figure S4**. **Embryo lethality in two independent *faah3* mutant alleles is observed in dissected siliques.** The valves of fully expanded siliques (number 14-16 from the top) were stripped using Dumoxel Style 5 forceps under a Nikon SMZ645 dissection stereomicroscope and imaged using a QImaging MicroPublisher 5.0 RTV coupled to a Leica MZ125 dissection stereomicroscope. Scale bar=0.5mm. WT: wild-type (Col-0), *f3-1/+:* heterozygous *faah3-1* (SALK_082643), *f3-2/+*: heterozygous *faah3-2* (GABI_137D02), *ami1-1 tIII tV f1 f2 f4*: *ami1-1 toc64-III toc64-V faah1 faah2 faah4*, *ami1-1 tIII tV f1 f2 f3/+ f4: ami1-1 toc64-III toc64-V faah1 faah2 faah3-1/+ faah4.* Scale bar = 0.5mm.


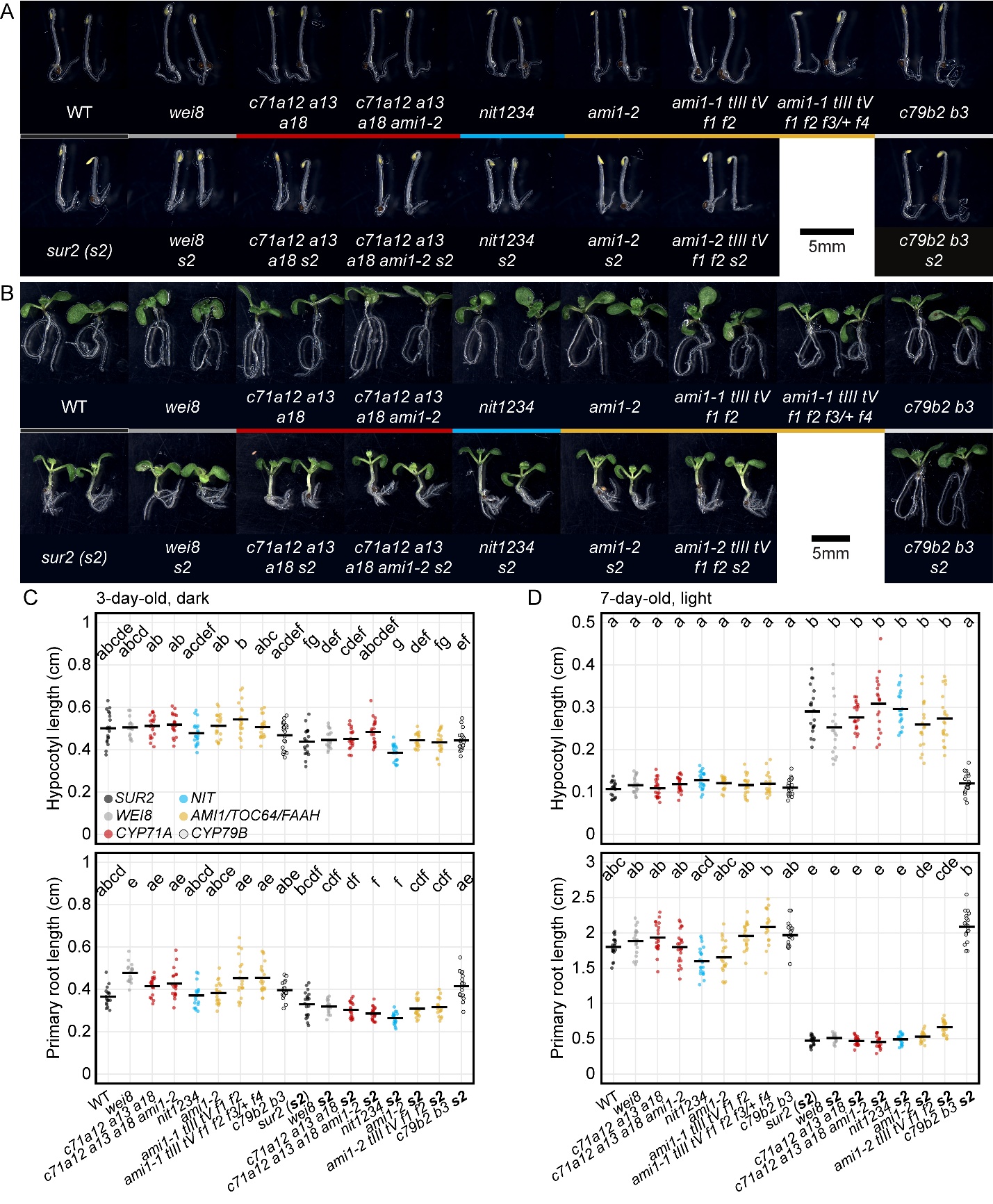


**Supplementary Figure S5**. **Mutants defective in the proposed IAOx pathway of auxin biosynthesis display no prominent growth defects**. (A, B) Seedlings were germinated on horizontal plates in the dark for three days (A) or under continuous light for seven days (B). (C, D) Hypocotyl- and root-length quantification of seedlings grown for three days in the dark (C) or for seven days under continuous light (D). Different letters denote statistically significant differences for α=0.05. Genotypes in WT or *sur2* background are color-coded by the mutated gene family: black (*SUR2*: WT and *sur2*), dark grey (*WEI8*: *wei8* and *wei8 sur2*), red (*CYP71A*: *c71a12a13a18*, *c71a12a13a18 ami1-2, c71a12a13a18 s2*, *c71a12a13a18 ami1-2 s2*), blue (*NIT*: *nit1234* and *nit1234 s2*), yellow (*AMI1/TOC64/FAAH*: *ami1-2*, *ami1-1 tIII tV f1 f2, ami1-1 tIII tV f1 f2 f3/+ f4, ami1-2 s2*, *ami1-1 tIII tV f1 f2 s2*), light grey and black border (*CYP79B:* *c79b2b3*, *c79b2b3 s2*). WT: wild-type (Col-0), *c71a12 a13 a18: cyp71a12 cyp71a13 cyp71a18, c71a12 a13 a18 ami1-2: cyp71a12 cyp71a13 cyp71a18 ami1-2, nit1234: nit1 nit2 nit3 nit4, ami1-1 tIII tV f1 f2: ami1-1 toc64-III toc64-V faah1 faah2, ami1-1 tIII tV f1 f2 f3/+ f4: ami1-1 toc64-III toc64-V faah1 faah2 faah3/+ faah4, s2: sur2, c79b2 b3: cyp79b2 cyp79b3.*


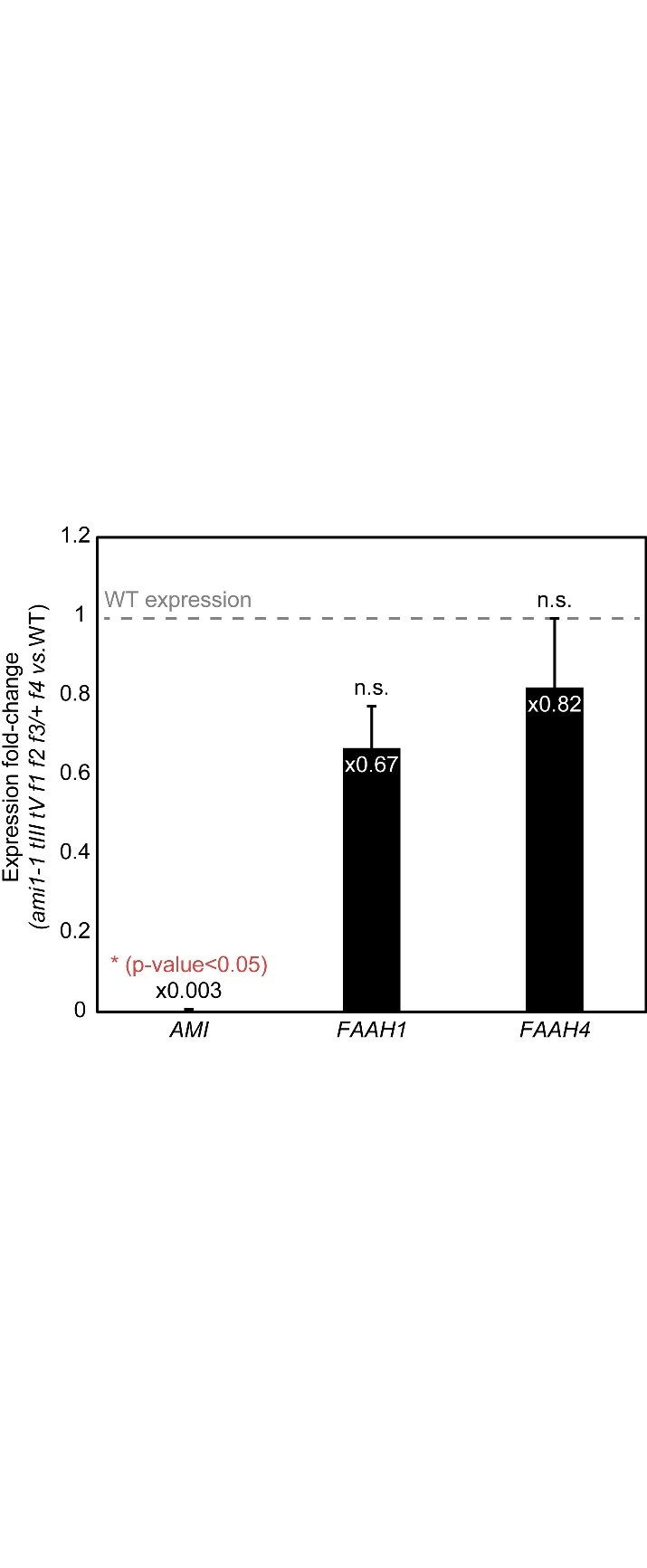


**Supplementary Figure S6. Expression analysis of intronic T-DNA alleles shows that *AMI1* is inactivated, but *FAAH1* and *FAAH4* are only partially knocked down.** RNA was extracted from ten-day old WT and *ami1-1 toc64-III toc64-V faah1 faah2 faah3/+ faah4* (noted as *ami1-2 tIII tV f1 f2 f3/+ f4*) whole seedlings grown under continuous light on horizontal AT plates and analyzed by RT-qPCR. Statistical analysis (t-Student, α=0.05) was performed using ΔΔCт values, and expression fold-change was calculated as 2^(-ΔΔCт). ΔΔCт *GENE1* = ΔCт (*GENE1*_*mutant*) - ΔCт (*GENE1*_WT). ΔCт_genotype1 = Cт (*GENE1*_genotype1) - Cт (*CBP20*_genotype1)*. CBP20: CAP-BINDING PROTEIN20* (*AT5G44200*) used as a housekeeping gene to normalize gene expression.


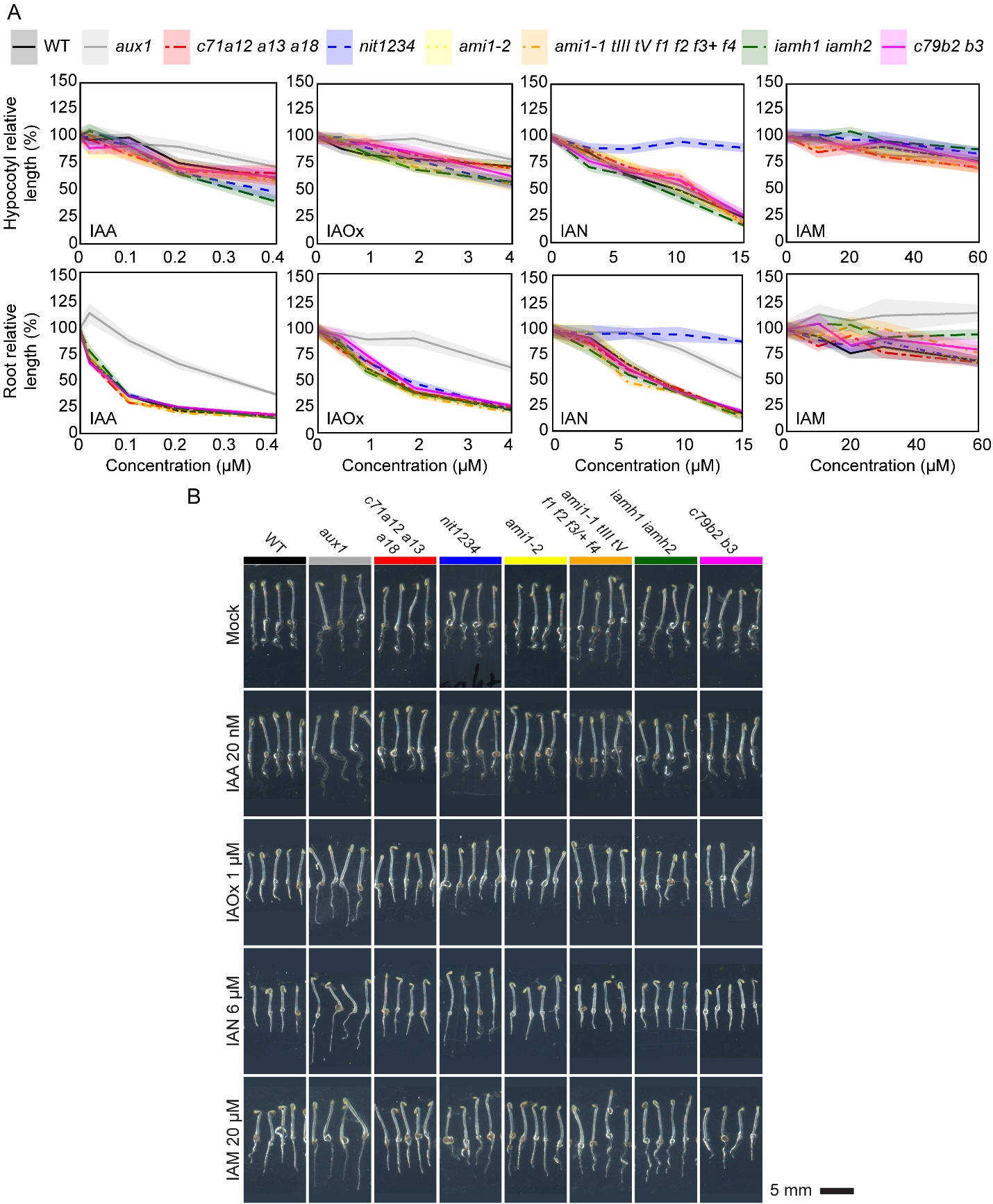


**Supplementary Figure S7**. **Phenotypes of dark-grown mutants impaired in the putative IAOx route challenge the established model of the IAOx pathway.** (A) WT and mutant lines were germinated on horizontal plates in the dark for three days in control media and in media supplemented with the indicated concentrations of IAA, IAOx, IAN and IAM (Supp. Table 2). For each treatment, the “0” concentration contains the equivalent concentration of DMSO as the highest concentration tested for a specific precursor. Root and shoot lengths were measured in ImageJ. Relative organ size at a given concentration for a specific genotype was calculated by dividing the organ size by that in the corresponding control ([metabolite]=0). Average relative organ sizes (lines) and confidence intervals (CI=95%, shades) were plotted using R studio. (B) Photographs of representative plants for one of the concentrations for each compound. These precursor concentrations were chosen as they produce similar organ sizes in the WT as 20nM IAA. Genotypes in WT or *sur2* background are color-coded by the mutated gene family: black (*SUR2*: WT and *sur2*), dark grey (*WEI8*: *wei8* and *wei8 sur2*), red (*CYP71A*: *c71a12a13a18*, *c71a12a13a18 ami1-2, c71a12a13a18 s2*, *c71a12a13a18 ami1-2 s2*), blue (*NIT*: *nit1234* and *nit1234 s2*), and yellow (*AMI1/TOC64/FAAH*: *ami1-2*, *ami1-1 tIII tV f1 f2, ami1-1 tIII tV f1 f2 f3/+ f4, ami1-2 s2*, *ami1-1 tIII tV f1 f2 s2*). WT: wild-type (Col-0), *aux1: aux1-7, c71a12 a13 a18: cyp71a12 cyp71a13 cyp71a18, nit1234: nit1 nit2 nit3 nit4, ami1-1 tIII tV f1 f2 f3/+ f4: ami1-1 toc64-III toc64-V faah1 faah2 faah3/+ faah4, iamh1 iamh2: iamh1-1 iamh2-2, c79b2 b3: cyp79b2 cyp79b3.*


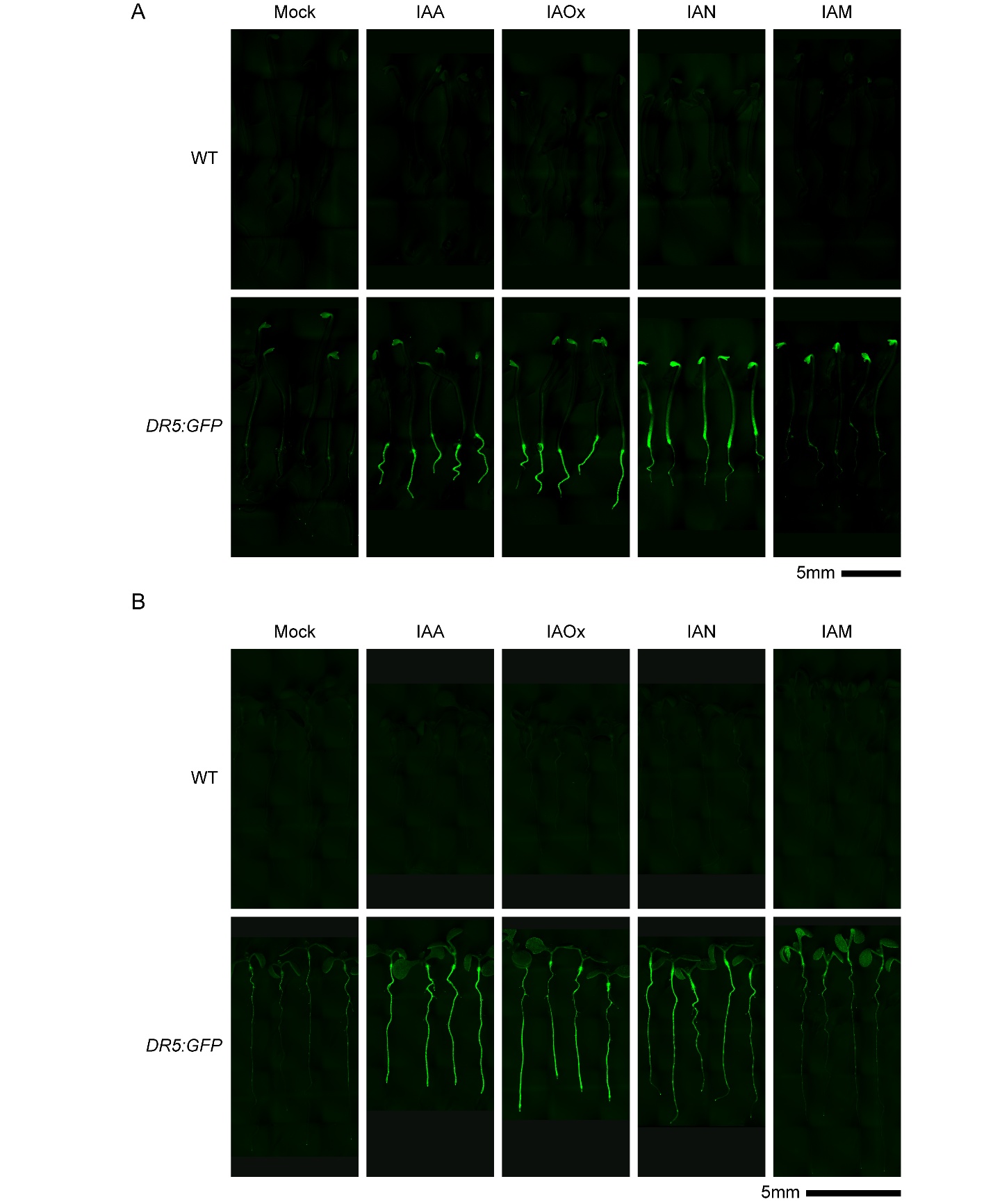


**Supplementary Figure S8**. **Exogenous application of putative IAOx intermediates induces activity of the auxin response reporter, *DR5:GFP***. (A,B) Three-day-old dark-grown seedlings (A) and five-day-old light-grown seedlings (B) were germinated on horizontal AT plates and transferred onto the indicated auxin precursors at concentrations empirically determined to be the lowest for seedlings to reach maximum change in organ size when germinated in the presence of that metabolite (Supplementary Fig. 6A). Dark experiment: 0.3µM IAA, 5µM IAOx, 20µM IAN, 50µM IAM, 0.25µM NAA. Light experiment: 3µM IAA, 20µM IAOx, 30µM IAN, 50µM IAM, 0.5µM NAA.


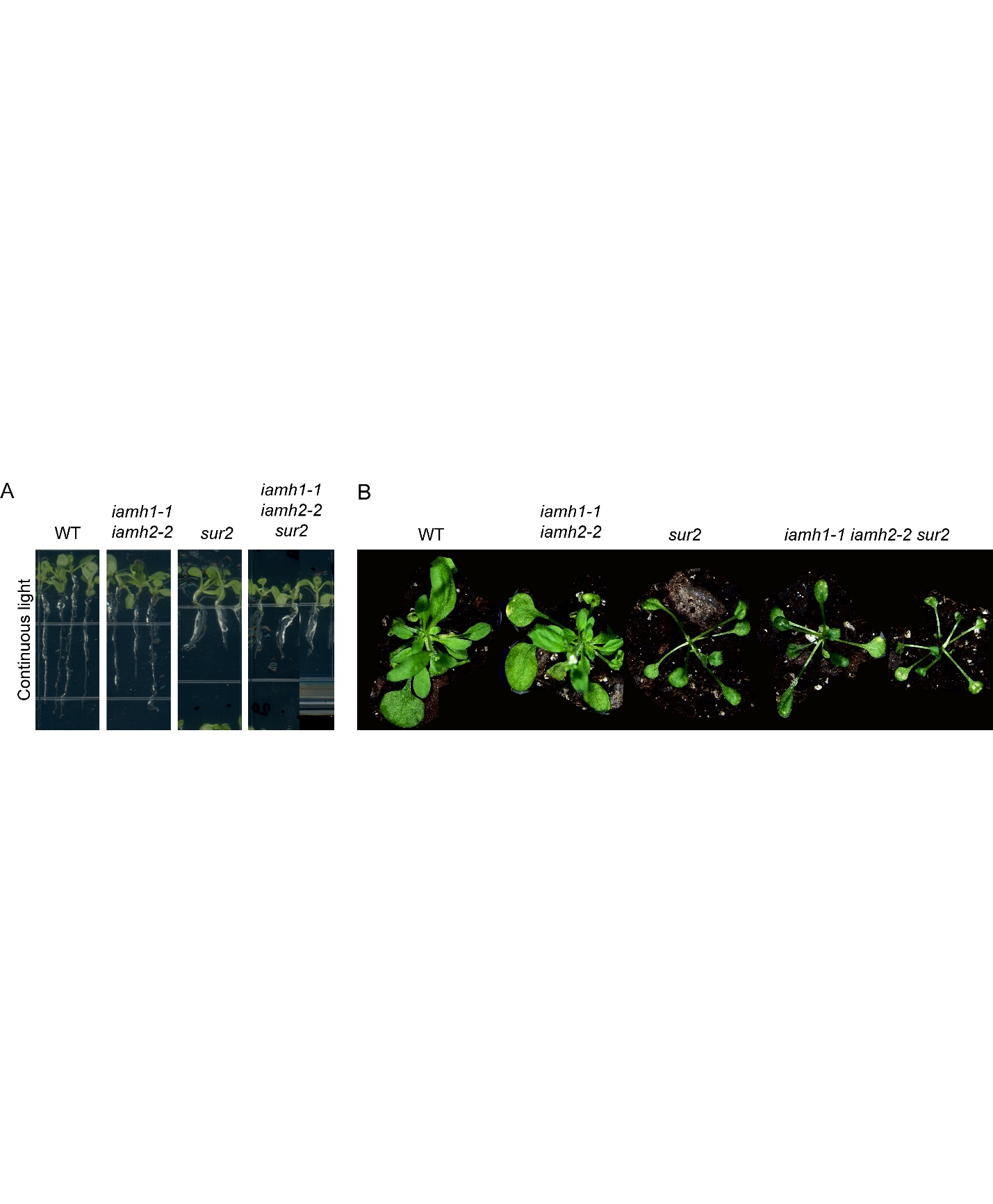


**Supplementary Figure S9. *iamh1 iamh2 sur2* mutants are indistinguishable from *sur2*.** (A) Seeds from two *iamh1/+ iamh2/+ sur2* F2 plants (MF2842 and MF2845) were germinated and grown under continuous LED light for ten days on horizontal AT plates. *iamh1-1* and *iamh2-2* are genetically linked, and therefore, one fourth of the F2 progeny plants were *iamh1-1 iamh2-2 sur2*, as confirmed by genotyping. (B) Seedlings displayed in (A) were transferred to soil and grown for two additional weeks in long-day conditions (16-h light:8-h darkness) under white LED light prior to imaging. Plants that were genotyped as *iamh1 iamh2 sur2* triple mutants were confirmed by NcoI digestion for *iamh2-2* and by Sanger sequencing for *IAMH1* (Gao et al., 2020). Therefore, we concluded that *iamh1-1 iamh2-2* mutations fail to suppress *sur2* phenotype.


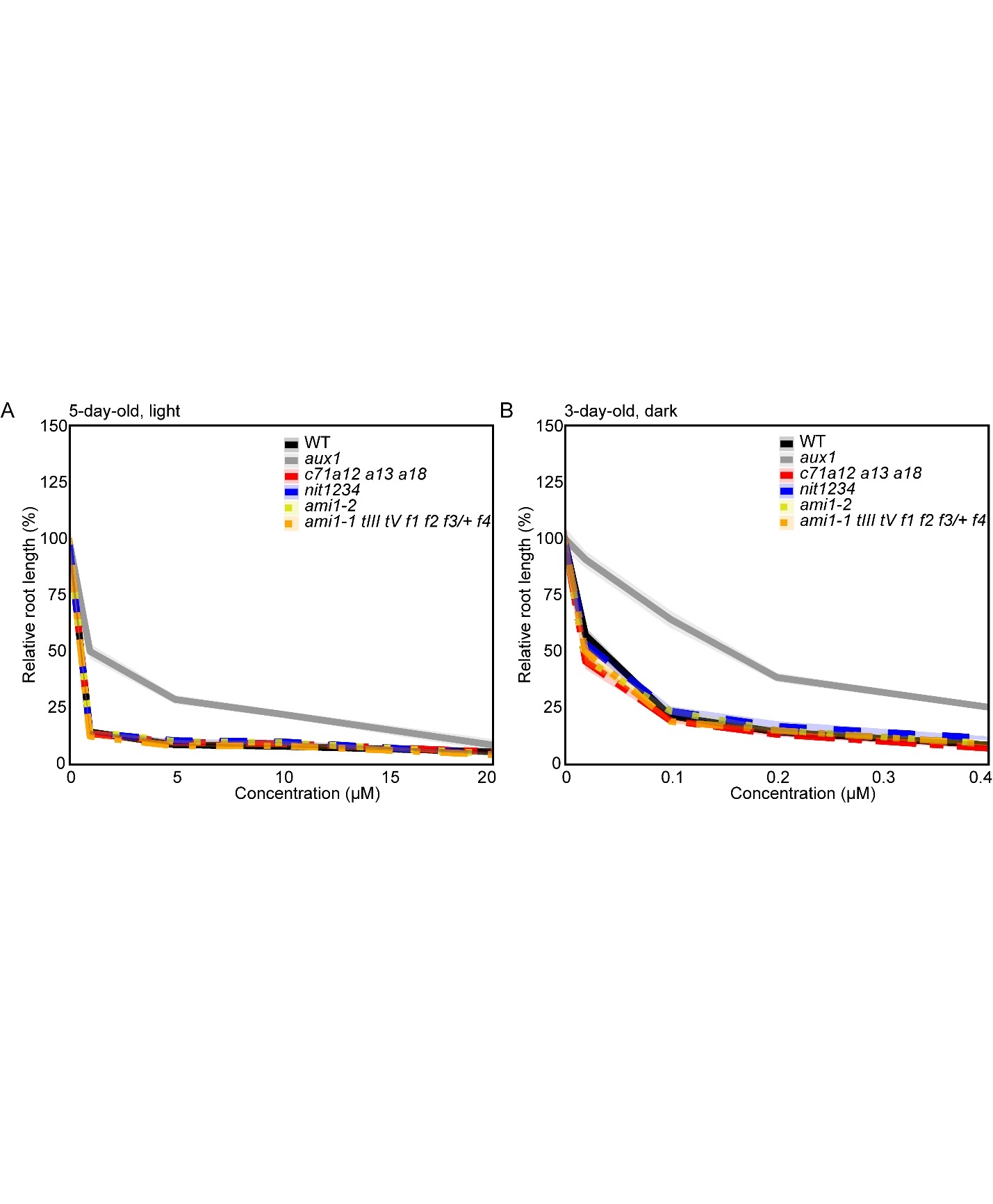


**Supplementary Figure S10**. **IAOx pathway mutants show normal root sensitivity to IAA.** (A, B) An independent repetition of the experiments displayed in Figure 3 (A) and Supp. Figure 5 (B) showing that IAOx mutants possess normal root sensitivity to IAA both in the light and in the dark. WT: wild-type (Col-0), *c71a12a13a18: cyp71a12 cyp71a13 cyp71a18, nit1234: nit1 nit2 nit3 nit4, ami1-1 tIII tV f1 f2 f3/+ f4: ami1-1 toc64-III toc64-V faah1 faah2 faah3/+ faah4.*


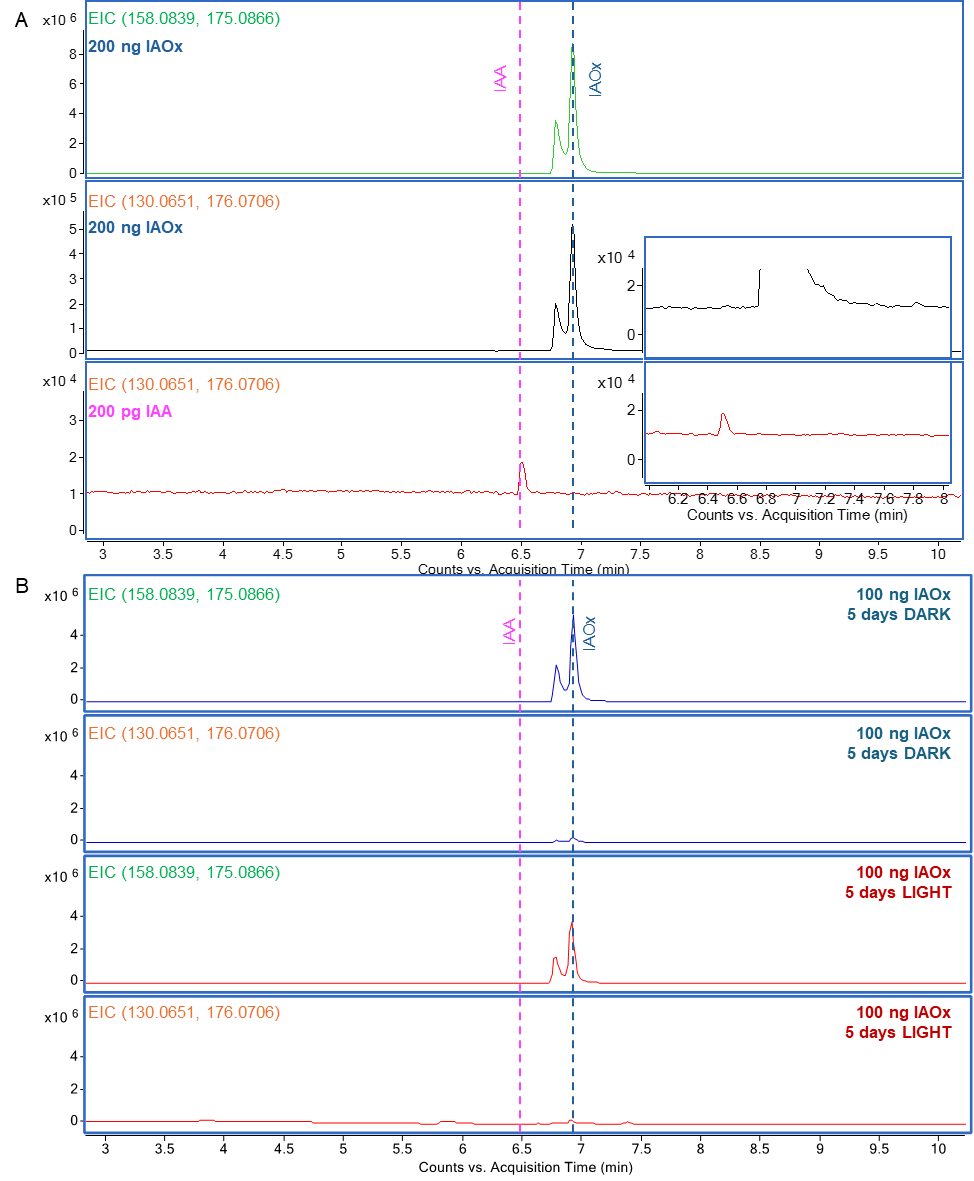


**Supplementary Figure S11. IAOx stock is not spontaneously converted into IAA**. (A) Extracted ion chromatograms (EICs) of IAOx and IAA standards. EIC of 158.0839 + 175.0866 was used to detect IAOx; EIC of 130.0651 + 176.0706 was used to detect IAA. The insert shows the bottom two EICs at the same y axis scale. (B) An aqueous solution of IAOx (10 ng/uL) was incubated in a 22 °C growth chamber under light or in the dark for 5 days, and analyzed by LC-MS using the same method as in A.


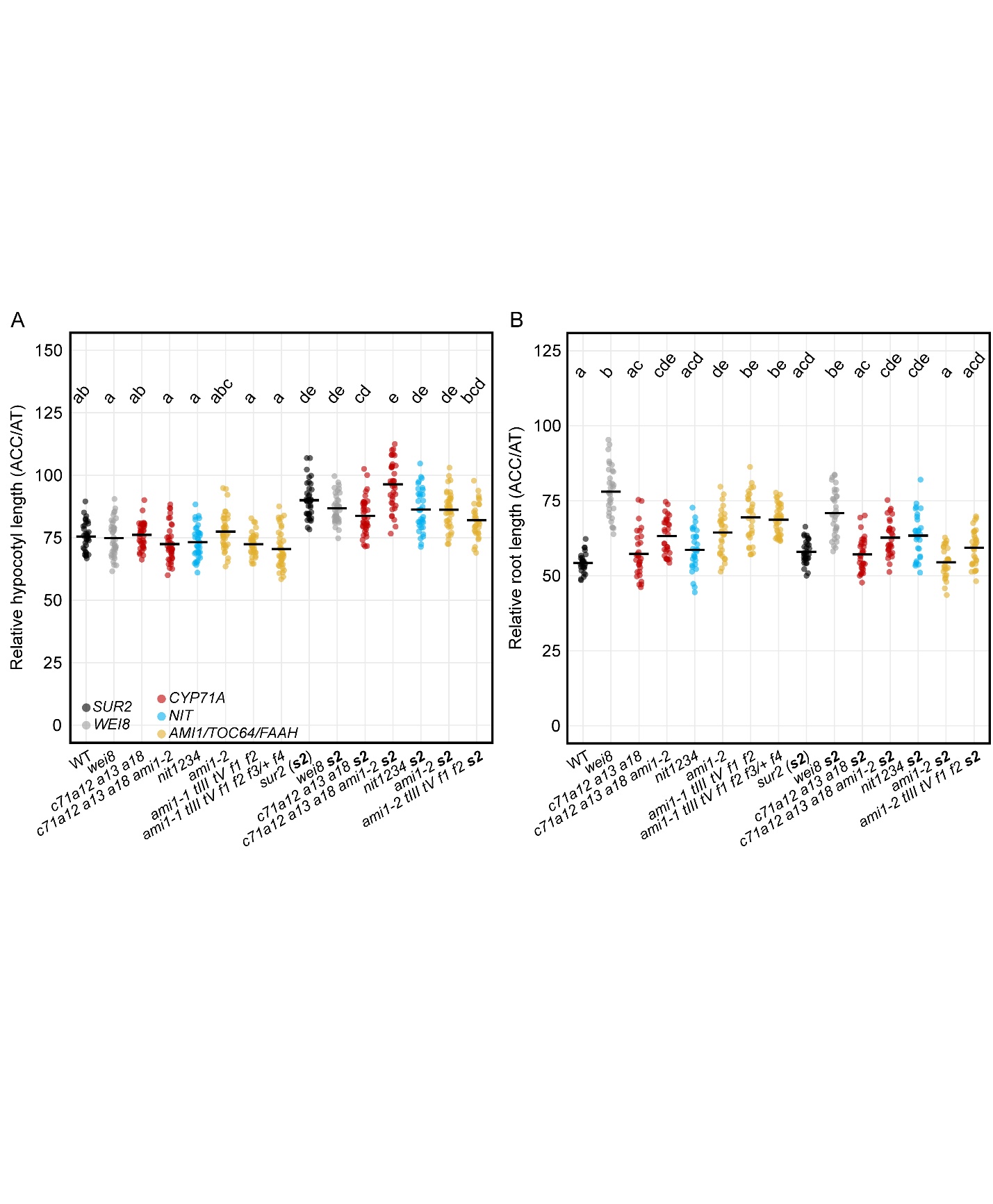


**Supplementary Figure S12**. **IAOx mutant hypocotyls and roots show WT-level of response to exogenous ACC.** Examples of other experimental repetitions quantifying relative growth of hypocotyls (A) and roots (B) in response to ACC show that the mild defects observed in *cyp71a12a13a18 s2* hypocotyls and *nit1234 s2* roots in Figure 5A-B are not consistently observed across repetitions and thus are unlikely to be biologically significant. Relative organ growth was calculated by dividing organ length of three-day-old, etiolated seedlings germinated on horizontal plates in the presence of the ethylene precursor ACC (0.2µM) by that in control media (AT). Different letters denote statistically significant differences for α=0.05. Genotypes in WT or *sur2* background are color-coded by the mutated gene family: black (*SUR2*: WT and *sur2*), dark grey (*WEI8*: *wei8* and *wei8 sur2*), red (*CYP71A*: *c71a12a13a18*, *c71a12a13a18 ami1-2, c71a12a13a18 s2*, *c71a12a13a18 ami1-2 s2*), blue (*NIT*: *nit1234* and *nit1234 s2*), and yellow (*AMI1/TOC64/FAAH*: *ami1-2*, *ami1-1 tIII tV f1 f2, ami1-1 tIII tV f1 f2 f3/+ f4, ami1-2 s2*, *ami1-1 tIII tV f1 f2 s2*). WT: wild-type (Col-0), *c71a12 a13 a18: cyp71a12 cyp71a13 cyp71a18, c71a12 a13 a18 ami1-2: cyp71a12 cyp71a13 cyp71a18 ami1-2, nit1234: nit1 nit2 nit3 nit4, ami1-1 tIII tV f1 f2: ami1-1 toc64-III toc64-V faah1 faah2, ami1-1 tIII tV f1 f2 f3/+ f4: ami1-1 toc64-III toc64-V faah1 faah2 faah3/+ faah4, s2: sur2.*


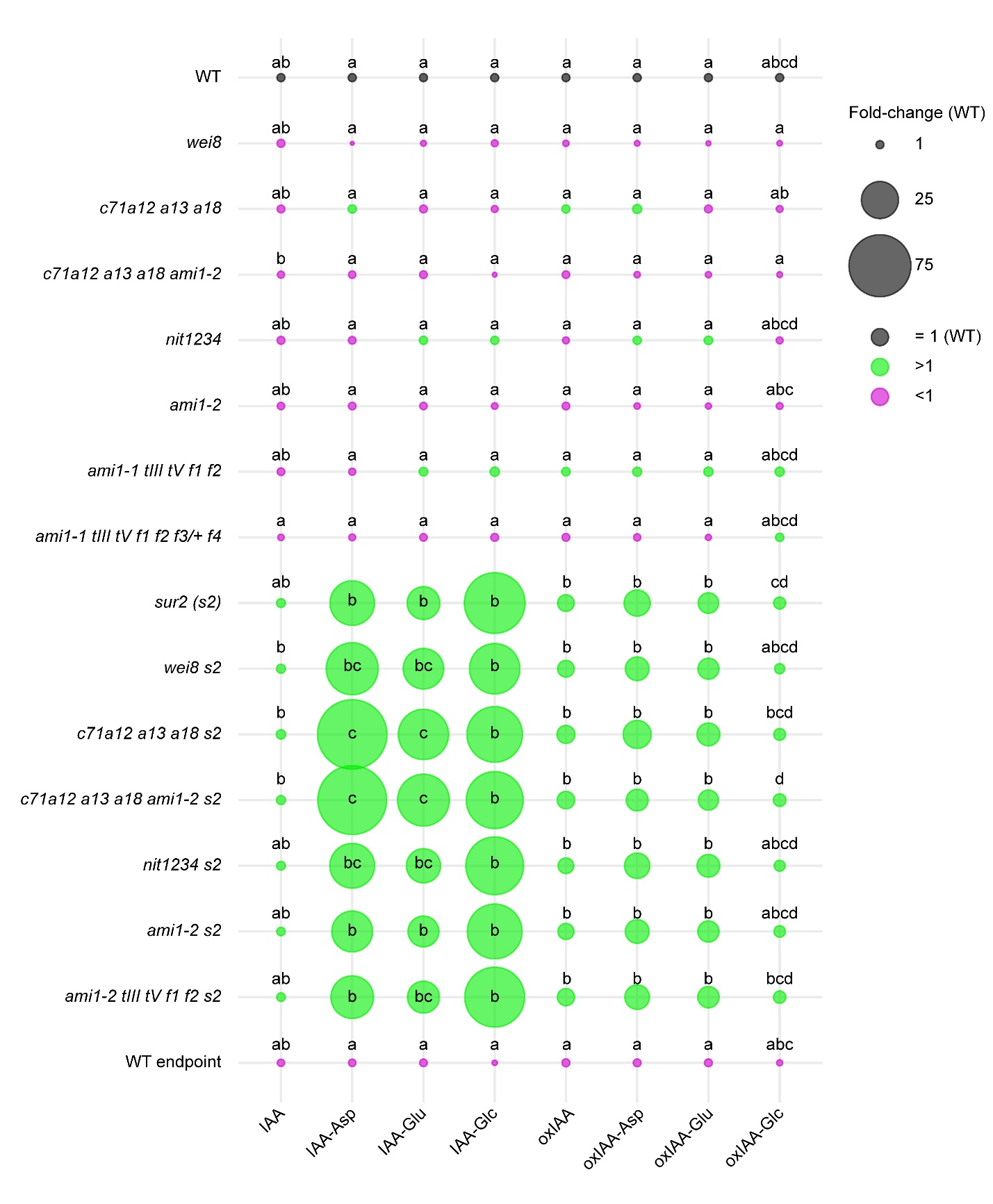


**Supplementary Figure S13**. **Metabolic quantification of IAA and its degradation products in IAOx shows major upregulation of homeostasis mechanisms leading to higher auxin degradation in *sur2***. Concentrations are normalized to WT values for each metabolite. Bubble sizes are proportional to the concentration fold changes for each mutant compared to WT. Fold change of >1 is shown in green, and that of <1 is in magenta. Different letters denote statistically significant differences between mean values of metabolite concentrations (log10 (pmol g FW^-1^)). IAA (indole-3-acetic acid) values are the same as the ones presented in Figure 7 and are included for reference. oxIAA: 2-oxindole-3-acetic acid. (ox)IAA-Asp: (ox)indole-3-acetyl-aspartate, (ox)IAA-Glu: (ox)indole-3-acetyl-glutamate, (ox)IAA-Glc: (ox)indole-3-acetyl-glucose. WT: wild-type (Col-0), *c71a12 a13 a18: cyp71a12 cyp71a13 cyp71a18, c71a12 a13 a18 ami1-2: cyp71a12 cyp71a13 cyp71a18 ami1-2, nit1234: nit1 nit2 nit3 nit4, ami1-1 tIII tV f1 f2: ami1-1 toc64-III toc64-V faah1 faah2, ami1-1 tIII tV f1 f2 f3/+ f4: ami1-1 toc64-III toc64-V faah1 faah2 faah3/+ faah4, s2: sur2.*


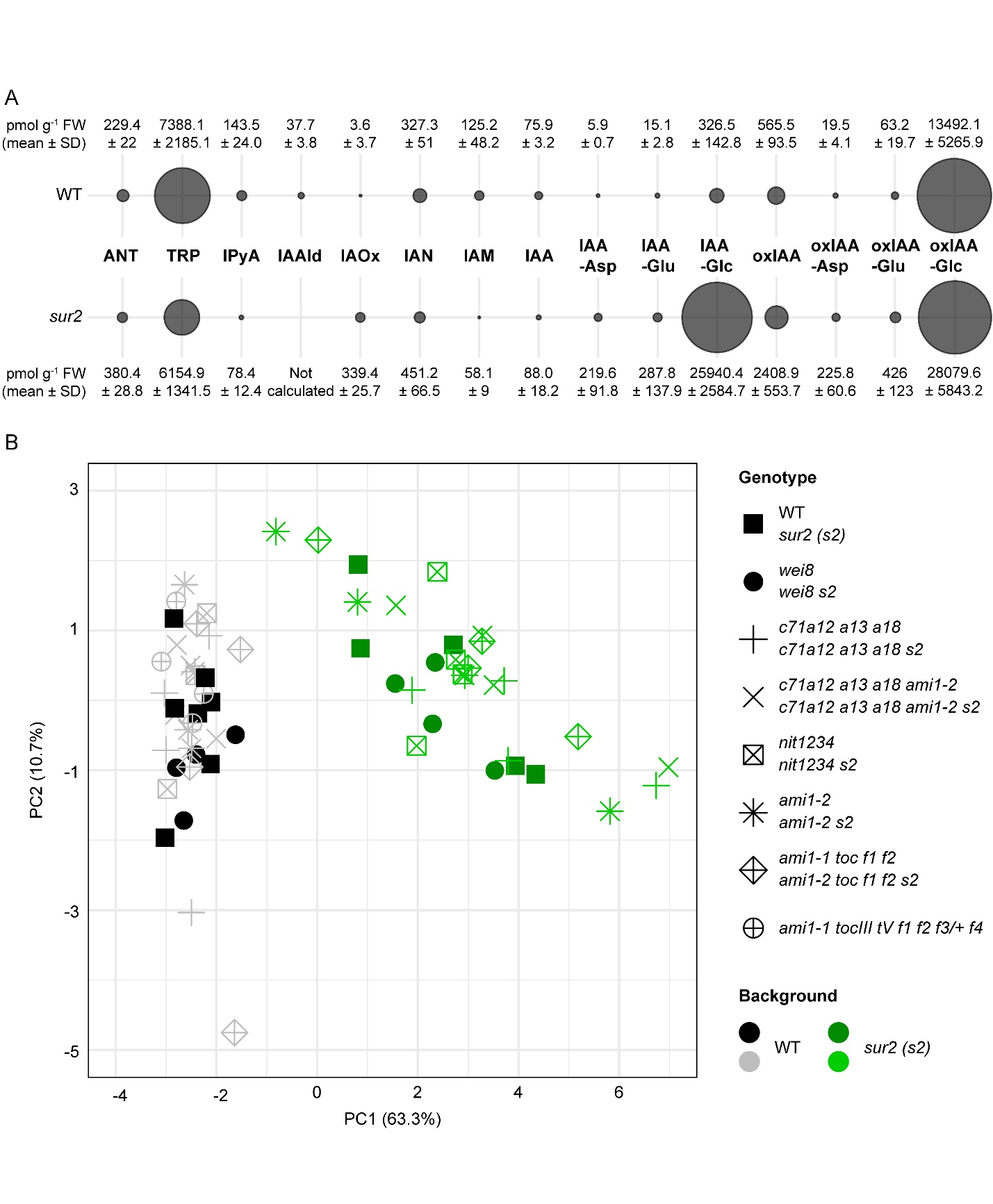


**Supplementary Figure S14**. ***sur2* background is the only mutation tested that prominently impacts the Arabidopsis metabolic profiles in seedlings**. (A) Absolute values of metabolites measured in pmol/g FW of WT (Col-0) and *sur2*. (B) Principal Component Analysis of metabolic profiles for all metabolites (not including IAAld or TAM) in the mutants. <LOD values were substituted by 0, and NQ were substituted by the average value of the other replicates when significantly different from 0. ANT: anthranilate, TRP: tryptophan, IPyA: indole-3-pyruvic acid, IAAld: indle-3-acetaldehyde, IAOx: indole-3-acetaldoxime, IAN: indole-3-acetonitrile, IAM: indole-3-acetamide, IAA: indole-3-acetic acid, oxIAA: 2-oxindole-3-acetic acid, (ox)IAA-Asp: (ox)indole-3-acetyl-aspartate, (ox)IAA-Glu: (ox)indole-3-acetyl-glutamate, (ox)IAA-Glc: (ox)indole-3-acetyl-glucose. WT: wild-type (Col-0), *c71a12 a13 a18: cyp71a12 cyp71a13 cyp71a18, c71a12 a13 a18 ami1-2: cyp71a12 cyp71a13 cyp71a18 ami1-2, nit1234: nit1 nit2 nit3 nit4, ami1-1 tIII tV f1 f2: ami1-1 toc64-III toc64-V faah1 faah2, ami1-1 tIII tV f1 f2 f3/+ f4: ami1-1 toc64-III toc64-V faah1 faah2 faah3/+ faah4, s2: sur2.*


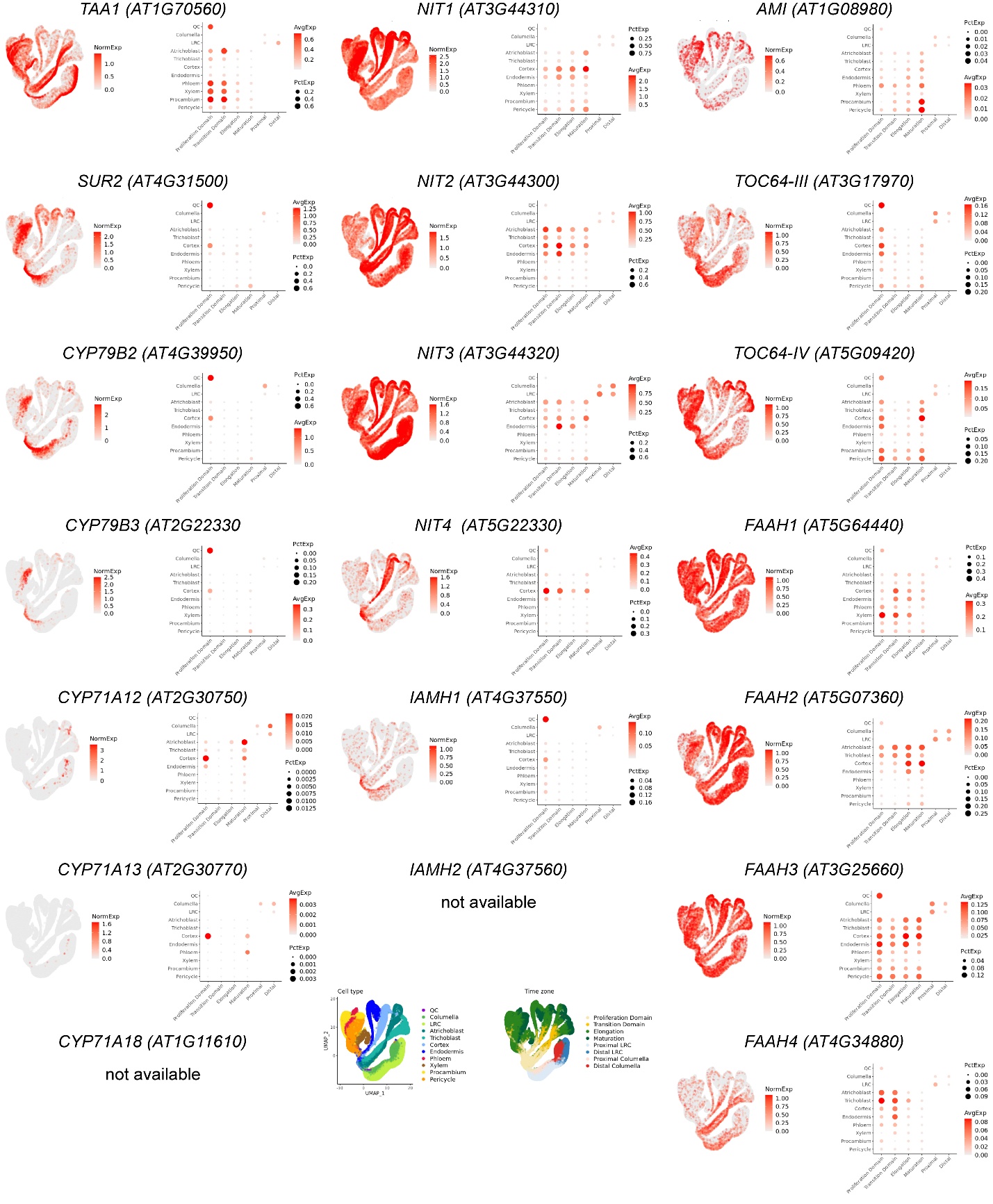


**Supplementary Figure S15. Single-cell gene expression analysis shows an overlap in the cortex cells, but not in the quiescent center (QC), between the gene families studied in this work.** Cells were collected from 5-day-old Arabidopsis WT roots from seedlings grown under long-day conditions. Data obtained from Nolan et al., 2023 and visualized using *Arabidopsis* Root Virtual Expression eXplorer (ARVEX; https://shiny.mdc-berlin.de/ARVEX/). NormExp: Normalized gene expression. AvgExp: Averaged normalized gene expression value in specific time zone & cell type. PctExp: Percentage of cells that the gene is expressed in specific time zone & cell type. Quantile cutoff (0.1-0.9): minimum and maximum cutoff values specified as quantiles for non-zero normalized gene expression. LRC: lateral root cap.
