# Supplementary material for: The CYP71A, NIT, AMI, and IAMH gene families are dispensable for indole-3-acetaldoxime-mediated auxin biosynthesis in Arabidopsis
Source: Plant Cell. 2025 Oct 15;37(11):koaf242. doi: 10.1093/plcell/koaf242 (PMC12586335; doi:10.1093/plcell/koaf242)
Supplement: koaf242_Supplementary_Data [file koaf242_supplementary_data.zip › Supp. Tables final.pdf]

Supplementary Table 1. Mutant lines used or generated in this work and their stock numbers.

Mutant line	Source
wei8-1	CS16407; Stepanova et al., 2008
cyp71a12 cyp71a13 cyp71a18	This work
cyp71a12 cyp71a13 cyp71a18 ami1-2	This work
nit1 nit2 nit3 nit4	This work
ami1-2	SALK_019823; Pérez-Alonso et al., 2020
ami1-1 toc64-III toc64-V faah1 faah2	This work
ami1-1 toc64-III toc64-V faah1 faah2 faah3/+ faah4	This work
sur2 DR5:GUS	CS16401; Stepanova et al., 2005
wei8-1 sur2	CS16437; generated in Stepanova et al., 2008, characterized in this work
cyp71a12 cyp71a13 cyp71a18 sur2	This work
cyp71a12 cyp71a13 cyp71a18 ami1-2 sur2	This work
nit1 nit2 nit3 nit4 sur2	This work
ami1-2 sur2	This work
ami1-2 toc64-III toc64-V faah1 faah2 sur2	This work

Supplementary Table 2. Primer combinations used for genotyping and PCR product size.

Allele	Mutant line	Mutant allele	WT allele
wei8-1	Act tag T-DNA (Stepanova et al., 2008)	DWLB1+7056-R1	70560-F8+70560-R1
		~800 bp	850 bp
aux1-7	3218G>A (Pickett et al., 1990)	AUX1_F+aux1_EcoRV_R, then digest with EcoRV	
		330 bp	355 bp
cyp79b2	Pooled SALK collection (Zhao et al., 2002)	79b2-F+JMLBa	79b2-F+79b2-R
		>803 bp	803 bp
cyp79b3	Pooled SALK collection (Zhao et al., 2002)	JMLBa+79b3-R	79b3-F+79b3-R
		>378 bp	378 bp
sur2	SALK_028573 (Alonso et al., 2003; Stepanova et al., 2005)	CYP83B1-F1+JMLBa	CYP83B1-F1+CYP83B1-R1
		~1000 bp	803 bp
cyp71a12	*cyp71a12*^{TALEN}*/a13n* (Müller et al., 2015)	A 5 bp deletion genetically linked to *cyp71a13*	
cyp71a13	SALK_105136 (this work; Alonso et al., 2003)	JMLB1+71A13-R2a	JMLB1+71A13-F2a+71A13-R2a
		>839 bp	1163 bp
cyp71a18	WiscDsLox297300_18A (this work; Woody et al., 2007)	WiscLB1+71A18-R1	MF_71A18-F2+71A18-R1
		>341 bp	778 bp
nit1	*NIT2/NIT1*^{CRISPR} (this work)	NIT2NewFor + NIT1NewRev 692 bp	NIT1FlankFor+NIT1NewRev
			586 bp
nit2			NIT2NewFor+NIT2FlankRev
			669 bp
nit3	SALK_015941 (this work; Alonso et al., 2003)	NIT3-F2+JMLBa	NIT3-F2+NIT3-R2
		>254 bp	801 bp
nit4		NIT4-F1+JMLBa	NIT4-F1+NIT4-R1

	SALK_016289 (this work; Alonso et al., 2003)	>501 bp	832 bp
ami1-1/ami1-2	*ami1-1*: SALK_069970 (Alonso et al., 2003; Aronsson et al., 2007; Pérez-Alonso et a., 2020)	JMLB1+AMI1-R2	AMI1-F2+AMI1-R2
	ami1-2: SALK_019823 (Alonso et al., 2003; Pérez-Alonso et a., 2020)	>838 bp for *ami1-1* and >400 bp for *ami1-2*	916 bp
toc64-III	*toc64-III-1* (Rios et al., 2002; Aronson et al., 2007)	mut 64-III-LB+64-III-R(PJ)	64-III-F+64-III-R(PJ)
		>700 bp	642 bp
toc64-V	Garlic_565_D12 (Session et al., 2002; Aronson et al., 2007)	SAIL-LB3+64-V-R	64-V-F+64-V-R
		>200 bp	588 bp
faah1	SALK_095108 (Alonso et al., 2003; Wang et al., 2008; Keereetaweep et al, 2013)	FAAH1-F+JMLBa	FAAH1-F+FAAH1-R
		>500 bp	887 bp
faah2	SALK_011213 (Alonso et al., 2003; Keereetaweep et al, 2013)	JMLB1+FAAH2-R	FAAH2-F+FAAH2-R
		>520 bp	652 bp
faah3-1	SALK_082643 (this work; Alonso et al., 2003)	FAAH3-F+JMLB1	FAAH3-F+FAAH3-R 852 bp
		>450 bp	
faah3-2	GABI_137D02 (this work; Rosso et al., 2003)	DWLB1+FAAH3-R	
		>450 bp	
faah4	SALK_029383 (this work; Alonso et al., 2003)	JMLBa+FAAH4-R2	FAAH4-F2+FAAH4-R2
		>700 bp	690 bp
iamh1	*iamh1-1 iamh2-2*[CRISPR] (Gao et al., 2020)	iamh1-1_F+iamh1-1_R, then digest with NcoI	
		1500 bp	600bp+900 bp
iamh2	*iamh1-1 iamh2-2*[CRISPR] (Gao et al., 2020)	SNP genetically linked to *iamh1-1*. DNA sequencing.	

Supplementary Table 3. Primer sequences used for mutant genotyping.

Target	Name	DNA sequence
T-DNA LB	JMLB1	GGCAATCAGCTGTTGCCCGTCTCACTGGTG
	JMLBa	CTTTGACGTTGGAGTCCACGTTC
	SAIL-LB3	TAGCATCTGAATTTCATAACCAATCTCGATACAC
	WISC-LB1	AACGTCCGCAATGTGTTATTAAGTTGTC
	TOC64-III-1 LB	GTTGACAGACTGCCTAGCATTTGAGTG
	DWLB1	CATACTCATTGCTGATCCATGTAGATTTCC
TAA1	70560-F8	CATCAGAGAGACGGTGGTGAAC
	70560-R1	GCTTTTAATGAGCTTCATGTTGG
AUX1	AUX1_F	GAGAGTCTGAGTATGACAAATC
	aux1_EcoRV_R	TACATTGGTAACACTTGGCAAAGATA
C79B2	C79B2-F	AGTATCATGACCCAATCATCGAC

	C79B2-R	CCATATCGGCTAAGAAGGAC
C79B3	C79B3-F	GCAATCCACCAATATCCGTCAG
	C79B3-R	GTTCTATGCATGGACTCGTGG
SUR2	CYP83B1-F1	GAGACTCTTGACCCTAACCGC
	CYP83B1-R1	GCGAGTCCAGTCATGACGTCC
C71A13	71A13-F2a	ATGGATAGATGGGATCCGTGG
	71A13-R2a	GGCAAACATCGATACCAATGGC
C71A18	MF_71A18-F2	GATGAAGGTATTTCATTACTAAGCCC
	71A18-R1	GACGAAATCCGCTTTATGTTCGCC
NIT1	NIT1FlankFor	GGCTATGGTTGGAGTCTTGG
	NIT1NewRev	AGCCTAGATGTTTCAAACGGC
NIT2	NIT2NewFor	AGGTTTGGACACTGATCCGT
	NIT2FlankRev	TTGCATCAAGAGCTGACCTTT
NIT3	NIT3-F2	ATCCACCACCGGTTCGTTCTGC
	NIT3-R2	GATCCGATTCTAACGGATCCTG
NIT4	NIT4-F1	TTGGTTGCTCCACCCGTGAC
	NIT4-R1	GAAGGATAGTCTTTCCGACGAC
AMI1	AMI1-F2	TCCAATGGCTCAGAGCTTCG
	AMI1-R2	CCACATTAGCTTGGAGATGCG
TOC64-III	TOC64-III-F	CCAAAGCCATCACCCTCGAC
	TOC64-III-R(PJ)	GACATGACCTTATTTTTGACGCTACGCTGAC
TOC64-V	TOC64-V-F	CATGCTACTCTAGGTGTTTGCC
	TOC64-V-R	GTAACCTCCAGCAAAGAGGG
FAAH1	FAAH1-F	GCAATGCAATAGGATCTCTACGAC
	FAAH1-R	GAGGTATCACTGGAGCTGTC
FAAH2	FAAH2-F	CTTGGTATCTTTCTCTAGTGCCG
	FAAH2-R	GGTATGTCATTGAGCCTGCTG
FAAH3	FAAH3-F	GCTGTTGCAGCAAGGCAGTG
	FAAH3-R	CTCCAAATCCTTCTCCACGG
FAAH4	FAAH4-F2	CTGCGTCCGAGTTCATACCTG
	FAAH4-R2	GGATACCCTCCAATGGCTAGG
IAMH1	iamh1-1_F	GATGACGCCAAGCGTGTAAGC
	iamh1-1_R	CTGGGAATTCAGAGGTAAGCAC
IAMH2	iamh2_F	GCATATAGGTAAAAGTTTCTTGGTGATTA
	iamh2_R	TCTGCTTGACAAAAAACAGGATGA

Supplementary Table 4. Concentrations (µM) of auxin biosynthesis precursors used in assays shown in Figure 3 (A) and Supp. Figure 6 (B).

A) Continuous light (5 days)

IAOx	40	20	10	5	Mock (DMSO like in 40 µM)
IAN	30	25	20	15	Mock (DMSO like in 30 µM)

IAM	60	30	20	10	Mock (DMSO like in 60 µM)
IAA	20	10	5	1	Mock (DMSO like in 20 µM)

B) Continuous darkness (3 days)

IAOx	4	2	1	0.5	Mock (DMSO like in 4 µM)
IAN	15	10	6	3	Mock (DMSO like in 15 µM)
IAM	60	30	20	10	Mock (DMSO like in 60 µM)
IAA	0.4	0.2	0.1	0.02	Mock (DMSO like in 0.4 µM)

Supplementary Table 5. Primer combinations used for expression analysis of intronic T-DNA alleles by RT-qPCR.

Target	Name	DNA sequence	Amplicon size
AMI1/ami1-1 (SALK_069970)	ami1-1_flk_F	GCTTCGACACAGTTGGATGGT	249 bp
	ami1-1_flk_R	CATTCTGTCCAATGTACTCTCCAA	
FAAH1/faah1 (SALK_095108)	faah1_flk_F	CAATCACGGTTGCAAAGTGGTG	164 bp
	faah1_flk_R	GCAAAGCTGGTACGAGTGTC	
FAAH4/faah4 (SALK_029383)	f4_flk_F	CAGGACAGTATTGGGCCGAT	245 bp
	f4_flk_R	ATGACAATAGCGCCTTCTCG	
CBP20 (*AT5G44200*)	*CBP20*_F	AATCGCCATGGAAGAGGAGAC	144 bp
	*CBP20*_R	GAATCGTGGGTTCTTCTCCGGTC	

The *faah3-1* intronic mutant allele was not included in this analysis as it is embryo-lethal in homozygotes and phenotypically similar to the *faah3-2* exonic allele (Supplementary Figure 4).
