# Supplementary material for: The CYP71A, NIT, AMI, and IAMH gene families are dispensable for indole-3-acetaldoxime-mediated auxin biosynthesis in Arabidopsis
Source: Plant Cell. 2025 Oct 15;37(11):koaf242. doi: 10.1093/plcell/koaf242 (PMC12586335; doi:10.1093/plcell/koaf242)
Supplement: koaf242_Supplementary_Data [file koaf242_supplementary_data.zip › Supplement References.docx]

**Supplement References**

**Camacho C, Coulouris G, Avagyan V, Ma N, Papadopoulos J, Bealer K, Madden TL** (2009) BLAST+: architecture and applications. BMC Bioinformatics **10**: 421

**Wang J, Chitsaz F, Derbyshire MK, Gonzales NR, Gwadz M, Lu S, Marchler GH, Song JS, Thanki N, Yamashita RA, et al** (2023) The conserved domain database in 2023. Nucleic Acids Res **51**: D384–D388
